# Dairy cattle herds mount a characteristic antibody response to highly pathogenic H5N1 avian influenza viruses

**DOI:** 10.1128/jvi.00621-25

**Published:** 2025-08-25

**Authors:** Lindsey R. Robinson-McCarthy, Holly C. Simmons, Aaron L. Graber, Carly N. Marble, Grace W. Graudin, Kevin R. McCarthy

**Affiliations:** 1 Center for Vaccine Research, University of Pittsburgh School of Medicine, Pittsburgh, Pennsylvania, USA; 2 Department of Microbiology and Molecular Genetics, University of Pittsburgh School of Medicine 12317 , Pittsburgh, Pennsylvania, USA; 3 University of Pittsburgh School of Medicine Program in Microbiology and Immunology 12317 , Pittsburgh, Pennsylvania, USA; St. Jude Children's Research Hospital, Memphis, Tennessee, USA

**Keywords:** virology, immunology, humoral immunity, veterinary immunology, surveillance studies

## Abstract

An unprecedented outbreak of a highly pathogenic avian influenza virus, H5 clade 2.3.4.4b, was reported in U.S. dairy cattle during the spring of 2024. It has now spread to hundreds of herds across multiple states. In humans, antibodies to the hemagglutinin (HA) protein confer the strongest protection against infection. Human herd immunity limits viral spread but also drives the emergence of antigenic variants that escape dominant antibody responses. We used store-bought milk to profile the collective H5N1 antibody response of dairy cattle herds. We detected HA binding antibodies in specific samples from states with recent/ongoing outbreaks. These antibodies present in milk neutralized replicating virus expressing dairy cattle HA and neuraminidase (NA). Despite originating from independent vendors, dairies/plants, geographic regions, and time, antibodies present in these samples are remarkably similar in activity and HA binding specificity. The dominant antibody response was clade 2.3.4.4b HA specific, followed by cross-reactivity with other H5s. Whether the uniformity of the response is a pathway to achieve herd immunity or an avenue for antigenic variants to rapidly escape remains to be seen.

**IMPORTANCE:**

Establishing human herd immunity ends pandemics. For influenza viruses, this immunity drives continued antigenic evolution that enables viruses to infect once-immune individuals. An outbreak of highly pathogenic avian influenza virus was detected in dairy cattle in 2024 and has spread rapidly across herds and states. We report approaches to assess dairy cattle herd immunity using store-bought milk samples. Across samples separated by geography and time, we find dairy cattle mount a strikingly similar antibody response that is strongest to the dairy cattle virus. Benchmarking immunity at this phase of the outbreak is important to understand either eradication or the emergence of antigenic variants that enable reinfection.

## INTRODUCTION

Highly pathogenic avian influenza (HPAI) viruses devastate commercial poultry flocks and can cause high rates of lethal disease in humans. This phenotype is dictated by the hemagglutinin protein (HA), which mediates cell entry (1). Influenza A HAs are classified numerically into 19 subtypes. A second glycoprotein, neuraminidase (NA), facilitates release and shares similar nomenclature. In 2020, an HPAI lineage from H5 clade 2.3.4.4b emerged and is responsible for the current panzootic involving six continents (2). An unprecedented outbreak in U.S. dairy cattle was detected in the spring of 2024 and has since spread to over 1,000 herds in 17 states as of July 2025 (3–5). Serologic data suggest that a far greater number of agricultural/veterinary workers have been infected than the 70+ documented human cases (6–9).

Historically, antigenically novel influenza viruses from animal reservoirs have triggered human pandemics (10, 11). Descendants of these viruses then circulate as endemic, seasonal viruses by acquiring mutations that confer resistance to antibody-mediated herd immunity elicited by previous infection and vaccination (12–16). An understanding of the dairy cattle antibody response to HPAI is needed to forecast the trajectory of the current outbreak and evaluate the consequences of viral antigenic variation. However, access to samples to widely profile immunity of cattle herds is limited.

To overcome these challenges, we developed approaches to profile antibody responses from cattle herds using store-bought milk. We detected H5 antibodies in specific samples from states with known H5N1 dairy cattle infections. Antibodies from milk can block cell entry and inhibit viral spread of a replicating virus expressing dairy cattle H5N1 HA and NA. We find that across samples from independent vendors, brands, dairies/plants, geographic regions, and time, antibodies present in these samples are remarkably similar in their pattern of HA reactivity. Clade 2.3.4.4b-specific antibodies dominate the response, with some cross-reactivity to other divergent H5s and less to HAs from different subtypes. We conclude that at the herd level, exposure to clade 2.3.4.4b viruses elicits this strikingly similar, stereotypic, antibody response. Whether this immunity will be sufficient to protect against current and future H5 viruses or drive the emergence of antigenic variants that escape it warrants increased surveillance.

## RESULTS

### Identification of H5-reactive antibodies in store-bought milk

Reports indicate that dairy cattle H5N1 viruses rapidly spread through herds (3, 4, 17). Following infection, antibodies to this virus should be present in the milk of lactating animals (18, 19). Given the number of cows likely exposed to H5N1 in affected herds (4, 5, 17, 20), we reasoned that H5-specific antibodies could be detected in store-bought milk. We obtained seven samples from California and two from Colorado in early October 2024 (milk 1–milk 7) (Table 1). These were chosen to encompass multiple milk fat percentages, dairies/processing plants, and expiration dates (as a proxy for processing dates). Milk samples were tested for reactivity with a recombinantly expressed, soluble, HA ectodomain trimer derived from A/dairy cow/Texas/24-008749-001/2024 (H5N1) in enzyme-linked immunosorbent assays (ELISAs). We used milk from Pennsylvania, where no cases had been reported at the time of purchase, as a negative binding control and an H5-binding human monoclonal antibody (mAb), FLD194 (21), as a positive binding control. One Colorado and one California milk sample demonstrated concentration-dependent H5 binding, which we defined as H5 antibody positive (Table 1; Fig. S1A).

**TABLE 1:** Store-bought milk samples contain H5 HA-reactive antibodies^*a*^*^,^*^*b*^

Sample	State	Brand	Plant	Milk fat	Expiration (mo/day/yr)	Ab positivity (ELISA)
1	PA	1	1	Whole	10/22/24	Neg
2	CA	2	2	Skim	10/22/24	Neg
3	CA	3	2	Skim	10/15/24	Pos
4	CA	4	3	Whole	10/17/24	Neg
5	CA	5	4	2%	11/21/24	Neg
6	CA*	–	–	Cream	–	Neg
7	CA*	–	–	Cream	–	Neg
8	CA*	–	–	2%	–	Neg
9	CO	6	5	2%	3/7/25	Neg
10	CO	7	6	Whole	10/21/24	Pos
11	CO	8	7	Whole	12/20/24	Neg
12	CO	9	7	Whole	12/2/24	Neg
13	CO	9	7	2%	12/20/24	Neg
14	CA	10	8	2%	12/10/24	Neg
15	CA	10	9	Whole	–	Neg
16	CA	8	10	–	–	Neg
17	CA	11	11	Whole	10/23/24	Neg
18	CA	8	3	Whole	10/31/24	Neg
19	MN	8	12	Whole	11/3/24	Neg
20	MI	12	13	Whole	12/18/24	Neg
21	OH	13	14	2%	10/29/24	Neg
22	CO	14	5	Skim	11/23/24	Pos
23	CO	14	5	Whole	11/28/24	Pos
24	CO	15	15	2%	11/20/24	Pos
25	CO	16	6	2%	11/18/24	Pos
26	CO	17	16	Whole	11/22/24	Pos
27	CO	18	6	2%	11/10/24	Pos
28	CO	7	6	Whole	11/20/24	Pos
29	PA	19	17	Whole	11/27/24	Neg
30	PA	1	1	Whole	11/29/24	Neg
31	MI	6	18	Whole	12/5/24	Neg
32	MI	6	18	1%	12/4/24	Neg
33	UT	20	19	Whole	12/2/24	Neg
34	MI	12	18	Whole	1/14/25	Neg
35	KS	3	20	Whole	1/17/25	Neg
36	CO	6	5	Half and half	3/9/25	Neg

^*a*^ Milk samples were collected from multiple states. “–” indicates that information was unavailable. *Samples 6–8 are presumed to be from California, but brand and processing plant information were not available. Antibody positivity was determined by ELISA with A/dairy cow/Texas/24-008749-001/2024 H5 HA (see Fig. S1).

^*b*^ Neg, negative; pos, positive.

To identify further H5 antibody positive samples, we purchased 24 additional milk products from states with reported dairy cattle H5N1 infections. This included milk from eight states bottled in 20 dairy/milk plants. A focused effort was made to acquire additional products from Colorado and California. Additional Pennsylvania negative controls were also obtained. Only seven samples, all from Colorado, had detectable levels of H5 HA reactivity (Table 1; Fig. S1B). Notably, all of the Colorado products with undetectable H5-binding antibodies were ultra-pasteurized, while those with detectable binding were not (Table S1). None of the additional California milk samples had detectable H5 HA binding antibodies. The one positive sample from California was collected before the scale of the outbreak was recognized and hundreds of herds were quarantined (22).

### Antibodies in milk have antiviral activity

**Fig 1:**
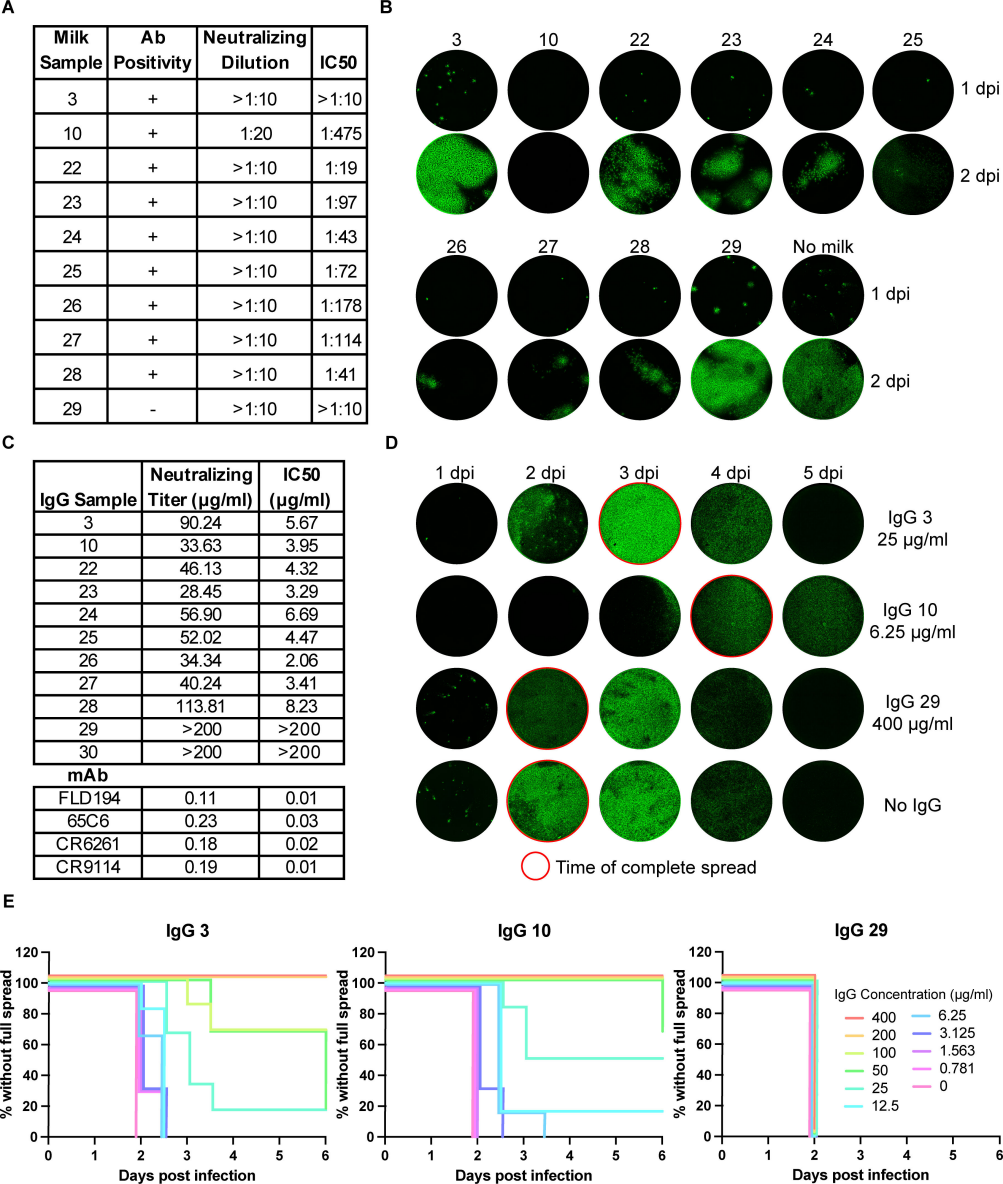
Antibodies in milk have antiviral activity. (**A**) Dilution at which each milk sample (Table 1) completely inhibited infection by rVSV-H5N1dc2024, and IC_50_ neutralizing titers for each milk sample. (**B**) Representative images of rVSV-H5N1dc2024-infected GFP-expressing cells at 1 and 2 days post-infection in the presence of each milk sample at a 1:10 dilution. (**C**) Neutralizing titers of purified IgG and mAbs (21, 24–26) for rVSV-H5N1dc2024. IgG sample numbers match the milk sample from which they were purified. (**D**) Representative images of rVSV-H5N1dc2024-infected cells in the presence of subneutralizing concentrations of purified IgG over 5 days. IgG concentration for each is indicated. Wells that have reached complete viral spread are outlined in red. Images taken at half-day intervals are not shown. (**E**) Protection from viral spread by purified milk IgG. Spread of rVSV-H5N1dc2024 infection in the presence of purified IgG was assessed twice per day over 5 days for each concentration of IgG. “Survival” was defined as conditions where infection had not spread throughout the well.

We used a replicating recombinant vesicular stomatitis virus (rVSV-H5N1dc2024) that expresses the H5 and N1 from A/dairy cow/Texas/24-008749-001/2024 in place of its glycoprotein (23) to assess the antiviral activity of antibodies in milk. This virus also expresses an enhanced green fluorescent protein (eGFP) reporter to track infection. We performed neutralization assays using the nine H5 antibody-positive milk samples and two Pennsylvania negative controls (Fig. 1A; Fig. S2). In these assays, the inoculum and milk remained on cells for the duration of the experiment. Milk concentrations did not exceed 10% vol/vol of the cell culture media. Only one, sample 10 from Colorado completely inhibited infection at dilutions of 1:10 and 1:20, although most had measurable half-maximal inhibitory concentration (IC_50_) values. However, for all H5 antibody-positive samples, we observed a concentration-dependent reduction in the number of infected foci and cell-cell spread over the course of 2 days (Fig. 1A and B). Antibodies in milk likely inhibit viral replication through multiple mechanisms, including directly blocking cell entry as seen by a reduction in the number of GFP foci, and by interfering with assembly or release of progeny virions as seen by the reduction in spread. In store-bought milk, which is pooled from potentially naive animals or ones in various states of convalescence, the concentrations of inhibitory antibodies appear relatively low.

We purified and concentrated IgG from these milk samples to further characterize their inhibitory functions. At the highest concentrations, antibodies from all nine H5 antibody-positive samples fully inhibited infection, while those from negative samples had no antiviral activity (Fig. 1C). We determined the minimal antibody concentration required to completely block infection and IC_50_ values (Fig. S2). Among our samples, these titers varied over a fourfold range. We then assessed the time needed for viruses to spread across the entire cell monolayer of an infected well. Inhibition of cell-cell spread occurred at greater dilutions than those required to fully prevent infection (Fig. 1D and E; Fig. S3).

### Dairy cattle mount a common antibody response to H5N1 viruses

We profiled the HA-reactivity of antibodies in milk using a panel of 83 recombinantly expressed, soluble HA ectodomain trimers (Table S2). These include the matched A/dairy cow/Texas/24-008749-001/2024 HA, a second clade 2.3.4.4b H5 HA, increasingly divergent H5 HAs from different clades/lineages, endemic human and animal H2, H1, H3, avian-origin H7, and human influenza B HAs. All nine ELISA-positive milk samples reacted most strongly with clade 2.3.4.4b HAs (Fig. S4). A minimal, non-specific, “background” level of HA reactivity was observed in our negative control samples.

**Fig 2:**
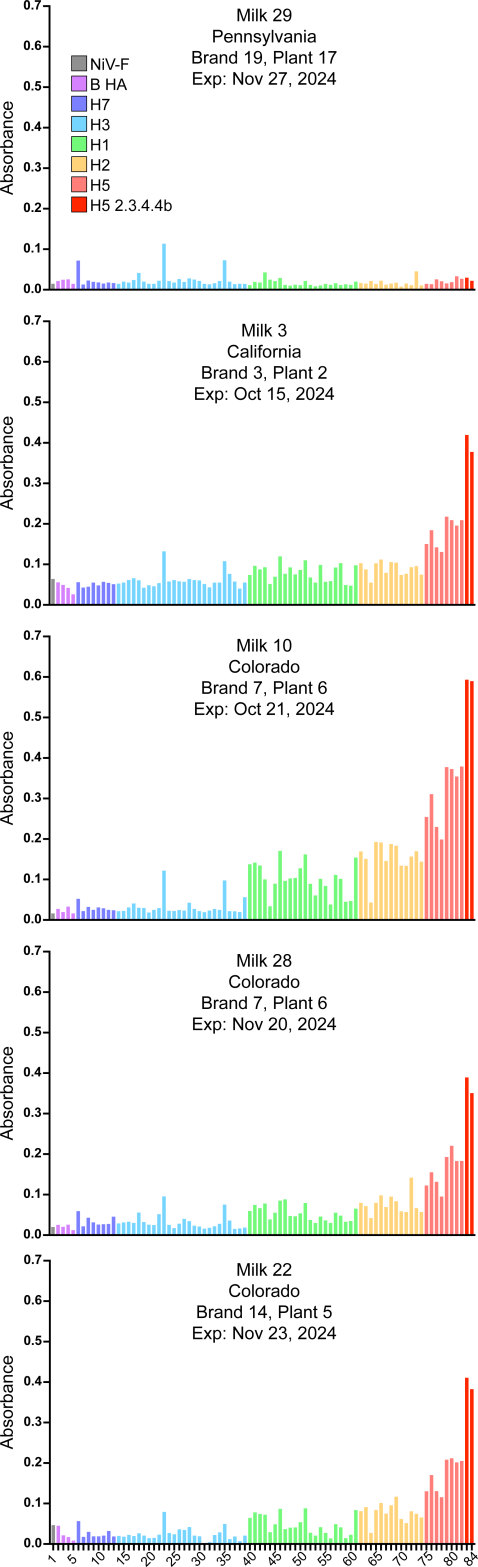
Breadth of HA reactivity of milk antibodies. Binding of antibodies in milk to recombinant full-length secreted ectodomain HA trimers from 83 unique isolates was assessed by ELISA. Nipah virus F protein (NiV-F) was included as a negative control. Bound antibody was detected using horseradish peroxidase-conjugated protein A/G. Bars are colored based on HA subtype, with H5 clade 2.3.4.4.b H5 in darker red for emphasis. The identity of each HA is provided in Table S2.

While the magnitude of reactivity to each HA differed across samples, we observed a strikingly consistent hierarchy of antibody responses across samples, time, plant/dairy, and states (Fig. 2). Binding was strongest to clade 2.3.4.4b HAs, followed by other H5s, H2s, and H1s. There was minimal reactivity with group 2 H3 and H7 HAs. The H5 specificity was more pronounced with milk as the analyte than with purified IgG, although both were strongly biased to group 1 HAs (Fig. S4 and S5).

**Fig 3:**
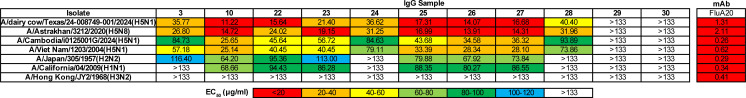
Purified IgG from milk reacts most strongly to clade 2.3.4.4.b H5 HAs. ELISA titrations were performed to determine the EC_50_ values for each purified IgG sample to H5, H2, H1, and H3 HAs. A/dairy cow/Texas/24-008749-001/2024 and A/Astrakhan/3212/2020 both belong to clade 2.3.4.4b. mAb FluA-20 (27) was included as a broadly binding control antibody.

We performed ELISA titrations with the purified IgGs because they could be used at higher concentrations and normalized for protein content (Fig. S6 and S7). Half-maximal effective concentrations (EC_50_) were lowest for clade 2.3.4.4b HAs (Fig. 3). EC_50_ values were approximately twofold higher for other H5s and nearly fivefold higher for H1s and H2s. Reactivity with H3 HA was minimal and similar to the reactivity of IgGs purified from the negative control Pennsylvania milk. Together, these data suggest that the strongest antibody response elicited by infection is H5 clade 2.3.4.4b dominant, sensitive to variation within H5 HAs, and strongly group 1 HA biased.

## DISCUSSION

An outbreak of HPAI in dairy cattle is unprecedented. The paucity of samples from confirmed H5N1 convalescent dairy cows has hindered the characterization of their antibody response to H5 clade 2.3.4.4b viruses. To overcome this challenge, we developed methods to profile the H5N1 antibody response at the scale of herd(s). Using store-bought milk, we created a snapshot of dairy cattle herd immunity elicited by a primary H5N1 exposure. Our “immunosurveillance” approach is generalizable and well suited for tracking the trajectory of exposure and immunity in this H5N1 outbreak or other endemic and emerging pathogens over time. Our ELISA-based assays and use of rVSV-H5N1dc2024 enable similar efforts to be performed in most molecular biology laboratories with limited need for specialized equipment or high biocontainment facilities.

Antibodies present in all of our H5 antibody-positive milk samples inhibit viral replication through multiple mechanisms but were present at relatively low concentrations. Milk from experimentally infected cows had maximal neutralizing titers between 8- and 25-fold higher than our most neutralizing bulk milk sample (18, 19). Using the eGFP reporter present in VSV-H5N1dc2024, we detected and quantified the activity of antibodies that prevent cell entry (neutralizing) and those that prevent cell-cell spread. Antibodies preventing cell-cell spread were inhibitory at subneutralizing milk/IgG concentrations. Given the high titers of infectious virus in the milk of actively infected cows prior to pasteurization (4, 18, 19), antibody concentrations in bulk milk tanks may be insufficient to fully neutralize H5N1 viruses and may therefore harbor infectious virus.

We found that the pattern of HA reactivity was remarkably similar across store-bought milk samples from multiple states, processing plants, brands, and time (same brand and plant). This pattern is defined by a strong clade 2.3.4.4b response, diminished binding to other H5s, and weaker binding to H2s and H1s (group 1 HAs). Binding to H3 or H7 (group 2 HAs) was limited or absent. Despite using a large array of recombinant HA proteins that include human and animal isolates, we did not find evidence of unreported, “cryptic,” dairy cattle infections from either H5 or other subtypes tested. Prior to this outbreak, infection of dairy cattle with influenza A viruses appears to be rare as no recall response was detected. Recent reports of dairy cattle infection with genetically distinct clade 2.3.4.4b viruses suggest that these viruses may be particularly well suited to infecting cows (28–30).

At the herd level, exposure to H5N1 reproducibly elicits a strikingly similar antibody response. Infection, or possibly vaccination with matched strains, is likely to elicit comparable responses in currently naive animals. Given the similarity of antibody responses across distinct herds and the relative magnitude of the H5 clade 2.3.4.4b-specific response, monitoring for antigenic variants that escape herd immunity is warranted.

## MATERIALS AND METHODS

### Milk samples

Commercially available, store-bought milk was obtained from grocery stores, convenience stores, and catered refreshments. The provenance of the milk was determined by the processing plant code. In instances where no plant code was provided, the dairy was confirmed to operate within the state of purchase. Milk brand and processing plant information was deidentified to adhere to the current Centers of Excellence for Influenza Research and Response Best Practice Guidelines for the Reporting of Milk Testing and Results (31). Samples 6–8 were obtained from the coffee station at the West Coast Retrovirus Meeting in Palm Springs, CA, USA, in early October 2024. These samples were assumed to originate in California, but further information about their provenance was not available. Full information for milk samples tested is provided in Table S1.

### Cells and viruses

BSRT7 cells (32) were maintained at 37°C and 5% CO_2_ in Dulbecco’s modified Eagle medium (DMEM, Thermo Fisher) supplemented with 10% fetal bovine serum (FBS) and penicillin/streptomycin (pen/strep, Thermo Fisher). 293F cells were maintained at 37°C with 8% CO_2_ in FreeStyle 293 Expression Medium (Thermo Fisher) supplemented with pen/strep.

rVSV-H5N1dc2024 was previously generated (23) and propagated on BSRT7 cells in viral growth medium (DMEM supplemented with 2% FBS, pen/strep, and 25 mM HEPES). rVSV-H5N1dc2024 was titered by plaque assay on BSRT7 cells as previously described (23).

### Plasmids

Synthetic DNA corresponding to the full-length ectodomain (FLsE) of influenza HAs was cloned into a pVRC vector modified to encode a C-terminal thrombin cleavage site, a T4 fibritin (foldon) trimerization tag, and a 6xHis tag (33, 34). Point mutations to stabilize HA as a prefusion trimer were added to specific sequences (annotated in Table S2), based on previously reported stabilizing mutations (35). Synthetic DNA corresponding to a prefusion stabilized Nipah virus F protein (NiV-F) (36) was cloned into pVRC as above. Accession numbers for all HA sequences are provided in Table S2.

Synthetic DNA corresponding to the heavy- and light-chain variable domains of antibodies FLD194 (21), 65C6 (24), CR9114 (25), CR6261 (26), and FluA-20 (27) was ordered from IDT and cloned into modified pVRC8400 plasmids (33) containing full-length human IgG1 heavy chains or human kappa or lambda light chains.

All plasmid sequences were verified through Sanger sequencing (Azenta) or whole plasmid nanopore sequencing (Plasmidsaurus).

### Monoclonal antibody production

Recombinant mAbs were produced as previously described (37) by transient transfection of heavy- and light-chain plasmids into 293F cells using polyethylenimine (PEI) transfection reagent. Five days post-transfection, supernatants were collected, clarified by low-speed centrifugation, and incubated overnight with protein A agarose resin (GoldBio) at 4°C. Resin was collected in a chromatography column and washed with one column volume of 10 mM Tris(hydroxymethyl)aminomethane (Tris), 150 mM NaCl at pH 7.5. mAbs were eluted in 0.1 M glycine (pH 2.5), which was immediately neutralized by 1 M Tris (pH 8.5). Antibodies were dialyzed against phosphate-buffered saline (PBS) at pH 7.4.

### Recombinant HA expression and purification

Recombinant HA FLsE and NiV-F were expressed and purified as previously described (37). Briefly, 293F cells were transiently transfected with FLsE-encoding plasmids using PEI. Transfection complexes were prepared in Opti-MEM and added to cells. Supernatants were harvested 4–5 days post-transfection and clarified by low-speed centrifugation. HA and NiV-F trimers were purified by passage over Co-NTA agarose (Clontech) followed by gel filtration chromatography on Superdex 200 (GE Healthcare) in 10 mM Tris-HCl and 150 mM NaCl at pH 7.5.

### H5-reactive antibody screening

Five hundred nanograms of recombinant HA FLsE per-well was adhered to 96-well plates (Corning) in PBS (pH 7.4) overnight at 4°C. HA-bound plates were washed with 0.05% Tween-20 in PBS (PBS-T) and blocked at room temperature for 1 hour in PBS-T with 2% bovine serum albumin (BSA). Blocking solution was removed, and twofold dilutions of milk in blocking solution were added to the wells. mAb FLD194 (21), diluted in fivefold dilutions starting at 500 ng/µL, was included on each plate as a positive binding control. Plates were then incubated for 1 hour at room temperature and then washed four times with PBS-T to remove milk/mAb. One hundred microliters of blocking buffer with peroxidase-conjugated recombinant protein A/G (Thermo Fisher) diluted 1:10,000 was added to each well and incubated for 1 hour at room temperature. Plates were washed three times with PBS-T and developed using 150 µL 1-Step ABTS substrate (Thermo Fisher). Following incubation at room temperature, horseradish peroxidase (HRP) reactions were stopped by the addition of 100 µL of 1% sodium dodecyl sulfate solution. Plates were read on a Molecular Devices SpectraMax 340PC384 Microplate Reader at 405 nm. All measurements were performed in single replicates. Background subtracted data were graphed using Prism 10 software (GraphPad).

### Antibody purification from milk

Milk or milk diluted one to one in 10 mM Tris-HCl, 150 mM NaCl at pH 7.5 was incubated with immobilized protein G resin (Thermo Scientific) overnight at 4°C. The resin was collected in a chromatography column and washed with at least two column volumes of 10 mM Tris-HCl, 150 mM NaCl at pH 7.5. Antibodies were eluted in 0.1 M glycine (pH 2.5), which was immediately neutralized by 1 M Tris (pH 8.5). Antibodies were then concentrated and dialyzed against PBS at pH 7.4.

### Milk neutralization assays

Neutralization assays were performed as previously reported, with some modifications (23). Milk samples were diluted in twofold serial dilutions in viral growth medium. One hundred plaque-forming units (PFU) of rVSV-H5N1dc2024 per well was added to diluted milk, and samples were incubated for 1 hour at room temperature. The milk:virus mixture was added to BSRT7 cells, which were incubated at 37°C. The milk:virus mixture was left on cells for the duration of the experiment. Cells were imaged for GFP expression 1 and 2 days post-infection using an EVOS automated fluorescence microscope (Therm Fisher) with a ×4 objective. Images were analyzed using Image J software (National Institutes of Health). Neutralizing titers are the dilution of milk that completely inhibited infection by 100 PFU of rVSV-H5N1dc2024. Fluorescent foci per well were counted from images taken 1 day post-transfection. Data were analyzed, and IC_50_ values were calculated using Prism 10 software (GraphPad).

### Purified IgG neutralization assays

Recombinant monoclonal antibodies and purified IgG from milk were diluted in serial twofold dilutions, and neutralization experiments were performed as described above. Antibody:virus mixture was maintained on the cells throughout the experiment. Cells were imaged twice per day over 5 days. Endpoint neutralizing titers are the concentration of IgG or mAb required to completely inhibit infection by 100 PFU of virus. Fluorescent foci per well were counted from images taken 1 day post-transfection. Data were analyzed using Prism 10 software (GraphPad) to determine IC_50_. Time to spread was determined for each well as the time point at which the virus had overtaken the entire well. Data were plotted as survival curves using Prism 10 software (GraphPad).

### Antibody binding breadth assay

One hundred nanograms of recombinant HA FLsE or NiV-F was adhered in duplicate to 96-well plates (Corning) in PBS at pH 7.4 overnight at 4°C. HA-bound plates were washed with 0.05% PBS-T and blocked at room temperature for 1 hour in PBS-T with 2% BSA. The blocking solution was removed and either diluted commercial milk samples or purified IgG from milk samples was added to each plate. Milk samples were diluted to 10% in blocking solution, and purified IgG was diluted to 1 mg/mL. Diluted milk or IgG was removed after 1 hour. Plates were washed three times with PBS-T and incubated with 1:10,000 peroxidase-conjugated recombinant protein A/G (Thermo Fisher) in blocking solution for 1 hour. Following secondary incubation, plates were washed three times with PBS-T and developed with 100 µL of 1-Step Slow TMB-ELISA Substrate Solution (Thermo Fisher) for 8 minutes, after which the reaction was stopped with 100 µL of 2M sulfuric acid. Absorbance values were measured using Molecular Devices SpectraMax 340PC384 Microplate Reader at 450 nm.

### Milk-derived IgG binding curve ELISAs

Five hundred nanograms of recombinant HA FLsE was adhered to 96-well plates (Corning) in PBS at pH 7.4 overnight at 4°C. HA-bound plates were washed with 0.05% PBS-T and blocked at room temperature for 1 hour in PBS-T with 2% BSA. The blocking solution was removed, and fivefold dilutions of purified IgG from milk were added to the wells. mAb FluA-20 was included on each plate as a positive control and to allow for normalization. The primary antibody was incubated for 1 hour and then removed. Plates were washed three times with PBS-T and incubated with 1:10,000 peroxidase-conjugated recombinant protein A/G (Thermo Fisher) in blocking solution for 1 hour. Following secondary incubation, plates were washed three times with PBS-T and developed with 100 µL of 1-Step Slow TMB-ELISA Substrate Solution (Thermo Fisher) for 8 minutes, after which the reaction was stopped with 100 µL of 2M sulfuric acid. Absorbance values were measured using Molecular Devices SpectraMax 340PC384 Microplate Reader at 450 nm. All measurements were performed in technical triplicate. Data were plotted and analyzed using Prism 10 software (GraphPad) to determine EC_50_.

## Data Availability

All data are reported here and in the supplemental material. No ancillary data sets were generated in this study. Data files are available upon request.

## SUPPLEMENTAL MATERIAL

Supplemental Material jvi.00621-25-s0001.pdf Fig. S1 to S7; Tables S1 and S2.

ASM does not own the copyrights to Supplemental Material that may be linked to, or accessed through, an article. The authors have granted ASM a non-exclusive, world-wide license to publish the Supplemental Material files. Please contact the corresponding author directly for reuse.

## References

[1] Horimoto T, Kawaoka Y. 1994. Reverse genetics provides direct evidence for a correlation of hemagglutinin cleavability and virulence of an avian influenza A virus. J Virol 68:3120–3128. doi:10.1128/JVI.68.5.3120-3128.1994 8151777 PMC236802

[2] Xie R, Edwards KM, Wille M, Wei X, Wong SS, Zanin M, El-Shesheny R, Ducatez M, Poon LLM, Kayali G, Webby RJ, Dhanasekaran V. 2023. The episodic resurgence of highly pathogenic avian influenza H5 virus. Nature 622:810–817. doi:10.1038/s41586-023-06631-2 37853121

[3] USDA. 2025. HPAI confirmed cases in livestock.

[4] Caserta LC, Frye EA, Butt SL, Laverack M, Nooruzzaman M, Covaleda LM, Thompson AC, Koscielny MP, Cronk B, Johnson A, Kleinhenz K, Edwards EE, Gomez G, Hitchener G, Martins M, Kapczynski DR, Suarez DL, Alexander Morris ER, Hensley T, Beeby JS, Lejeune M, Swinford AK, Elvinger F, Dimitrov KM, Diel DG. 2024. Spillover of highly pathogenic avian influenza H5N1 virus to dairy cattle. Nature 634:669–676. doi:10.1038/s41586-024-07849-4 39053575 PMC11485258

[5] Burrough ER, Magstadt DR, Petersen B, Timmermans SJ, Gauger PC, Zhang J, Siepker C, Mainenti M, Li G, Thompson AC, Gorden PJ, Plummer PJ, Main R. 2024. Highly pathogenic avian influenza A(H5N1) clade 2.3.4.4b virus infection in domestic dairy cattle and cats, United States, 2024. Emerg Infect Dis 30:1335–1343. doi:10.3201/eid3007.240508 38683888 PMC11210653

[6] CDC. 2025. H5 Bird Flu: current situation. Available from: https://www.cdc.gov/bird-flu/situation-summary/index.html

[7] Mellis AM, Coyle J, Marshall KE, Frutos AM, Singleton J, Drehoff C, Merced-Morales A, Pagano HP, Alade RO, White EB, et al.. 2024. Serologic evidence of recent infection with highly pathogenic avian influenza A(H5) virus among dairy workers - Michigan and Colorado, June-August 2024. MMWR Morb Mortal Wkly Rep 73:1004–1009. doi:10.15585/mmwr.mm7344a3 39509348 PMC11542770

[8] Shittu I, Silva D, Oguzie JU, Marushchak LV, Olinger GG, Lednicky JA, Trujillo-Vargas CM, Schneider NE, Hao H, Gray GC. 2025. A one health investigation into H5N1 avian influenza virus epizootics on two dairy farms. Clin Infect Dis 80:331–338. doi:10.1093/cid/ciae576 39658318

[9] Leonard J, Harker EJ, Szablewski CM, Margrey SF, Gingrich KF 2nd, Crossley K, Fletcher E, McCreavy CJ, Weis-Torres S, Wang D, Noble EK, Levine MZ, Pagano HP, Holiday C, Liu F, Jefferson S, Li Z-N, Gross FL, Reed C, Ellington S, Mellis AM, Olson SM. 2025. Notes from the field: seroprevalence of highly pathogenic avian influenza A(H5) virus infections among bovine veterinary practitioners - United States, September 2024. MMWR Morb Mortal Wkly Rep 74:50–52. doi:10.15585/mmwr.mm7404a2 39946278 PMC11824947

[10] Webster RG, Hinshaw VS, Bean WJ, Sriram G. 1980. Influenza viruses: transmission between species. Philos Trans R Soc Lond B Biol Sci 288:439–447. doi:10.1098/rstb.1980.0021 6103562

[11] Russell CA, Kasson PM, Donis RO, Riley S, Dunbar J, Rambaut A, Asher J, Burke S, Davis CT, Garten RJ, et al.. 2014. Improving pandemic influenza risk assessment. Elife 3:e03883. doi:10.7554/eLife.03883 25321142 PMC4199076

[12] Smith DJ, Lapedes AS, de Jong JC, Bestebroer TM, Rimmelzwaan GF, Osterhaus ADME, Fouchier RAM. 2004. Mapping the antigenic and genetic evolution of influenza virus. Science 305:371–376. doi:10.1126/science.1097211 15218094

[13] Westgeest KB, de Graaf M, Fourment M, Bestebroer TM, van Beek R, Spronken MIJ, de Jong JC, Rimmelzwaan GF, Russell CA, Osterhaus ADME, Smith GJD, Smith DJ, Fouchier RAM. 2012. Genetic evolution of the neuraminidase of influenza A (H3N2) viruses from 1968 to 2009 and its correspondence to haemagglutinin evolution. J Gen Virol 93:1996–2007. doi:10.1099/vir.0.043059-0 22718569 PMC3542130

[14] Sandbulte MR, Westgeest KB, Gao J, Xu X, Klimov AI, Russell CA, Burke DF, Smith DJ, Fouchier RAM, Eichelberger MC. 2011. Discordant antigenic drift of neuraminidase and hemagglutinin in H1N1 and H3N2 influenza viruses. Proc Natl Acad Sci USA 108:20748–20753. doi:10.1073/pnas.1113801108 22143798 PMC3251064

[15] Salk JE, Suriano PC. 1949. Importance of antigenic composition of influenza virus vaccine in protecting against the natural disease; observations during the winter of 1947-1948. Am J Public Health Nations Health 39:345–355. doi:10.2105/ajph.39.3.345 18124075 PMC1527846

[16] Tricco AC, Chit A, Soobiah C, Hallett D, Meier G, Chen MH, Tashkandi M, Bauch CT, Loeb M. 2013. Comparing influenza vaccine efficacy against mismatched and matched strains: a systematic review and meta-analysis. BMC Med 11:153. doi:10.1186/1741-7015-11-153 23800265 PMC3706345

[17] Peña-Mosca F, Frye EA, MacLachlan MJ, Rebelo AR, de Oliveira PSB, Nooruzzaman M, Koscielny MP, Zurakowski M, Lieberman ZR, Leone WM, Elvinger F, Nydam DV, Diel DG. 2025. The impact of highly pathogenic avian influenza H5N1 virus infection on dairy cows. Nat Commun 16:6520. doi:10.1038/s41467-025-61553-z 40664659 PMC12263987

[18] Halwe NJ, Cool K, Breithaupt A, Schön J, Trujillo JD, Nooruzzaman M, Kwon T, Ahrens AK, Britzke T, McDowell CD, et al.. 2025. H5N1 clade 2.3.4.4b dynamics in experimentally infected calves and cows. Nature 637:903–912. doi:10.1038/s41586-024-08063-y 39321846 PMC11754106

[19] Baker AL, Arruda B, Palmer MV, Boggiatto P, Sarlo Davila K, Buckley A, Ciacci Zanella G, Snyder CA, Anderson TK, Hutter CR, Nguyen TQ, Markin A, Lantz K, Posey EA, Kim Torchetti M, Robbe-Austerman S, Magstadt DR, Gorden PJ. 2025. Dairy cows inoculated with highly pathogenic avian influenza virus H5N1. Nature 637:913–920. doi:10.1038/s41586-024-08166-6 39406346 PMC11754099

[20] Oguzie JU, Marushchak LV, Shittu I, Lednicky JA, Miller AL, Hao H, Nelson MI, Gray GC. 2024. Avian influenza A(H5N1) virus among Dairy Cattle, Texas, USA. Emerg Infect Dis 30:1425–1429. doi:10.3201/eid3007.240717 38848249 PMC11210641

[21] Xiong X, Corti D, Liu J, Pinna D, Foglierini M, Calder LJ, Martin SR, Lin YP, Walker PA, Collins PJ, Monne I, Suguitan AL Jr, Santos C, Temperton NJ, Subbarao K, Lanzavecchia A, Gamblin SJ, Skehel JJ. 2015. Structures of complexes formed by H5 influenza hemagglutinin with a potent broadly neutralizing human monoclonal antibody. Proc Natl Acad Sci USA 112:9430–9435. doi:10.1073/pnas.1510816112 26170284 PMC4522749

[22] CFDA. 2025. H5N1 Bird Flu virus in Livestock, on California department of food and agriculture. Available from: https://www.cdfa.ca.gov/AHFSS/Animal_Health/HPAI.html

[23] Robinson-McCarthy LR, Zirckel KE, Simmons HC, Le Sage V, McCarthy KR. 2025. A replicating recombinant vesicular stomatitis virus model for dairy cattle H5N1 influenza virus glycoprotein evolution. J Virol 99:e0038925. doi:10.1128/jvi.00389-25:e0038925 40464562 PMC12282150

[24] Hu H, Voss J, Zhang G, Buchy P, Zuo T, Wang L, Wang F, Zhou F, Wang G, Tsai C, Calder L, Gamblin SJ, Zhang L, Deubel V, Zhou B, Skehel JJ, Zhou P. 2012. A human antibody recognizing a conserved epitope of H5 hemagglutinin broadly neutralizes highly pathogenic avian influenza H5N1 viruses. J Virol 86:2978–2989. doi:10.1128/JVI.06665-11 22238297 PMC3302345

[25] Dreyfus C, Laursen NS, Kwaks T, Zuijdgeest D, Khayat R, Ekiert DC, Lee JH, Metlagel Z, Bujny MV, Jongeneelen M, et al.. 2012. Highly conserved protective epitopes on influenza B viruses. Science 337:1343–1348. doi:10.1126/science.1222908 22878502 PMC3538841

[26] Ekiert DC, Bhabha G, Elsliger M-A, Friesen RHE, Jongeneelen M, Throsby M, Goudsmit J, Wilson IA. 2009. Antibody recognition of a highly conserved influenza virus epitope. Science 324:246–251. doi:10.1126/science.1171491 19251591 PMC2758658

[27] Bangaru S, Lang S, Schotsaert M, Vanderven HA, Zhu X, Kose N, Bombardi R, Finn JA, Kent SJ, Gilchuk P, Gilchuk I, Turner HL, García-Sastre A, Li S, Ward AB, Wilson IA, Crowe JE Jr. 2019. A site of vulnerability on the influenza virus hemagglutinin head domain trimer interface. Cell 177:1136–1152. doi:10.1016/j.cell.2019.04.011 31100268 PMC6629437

[28] USDA. 2025. The occurrence of another highly pathogenic avian influenza (HPAI) spillover from wild birds into dairy cattle. USDA Animal and Plant Health Inspection Service. Available from: https://www.aphis.usda.gov/sites/default/files/dairy-cattle-hpai-tech-brief.pdf

[29] USDA. 2025. APHIS identifies third HPAI spillover in dairy cattle. USDA Animal and Plant Health Inspection Service

[30] USDA. 2025. APHIS confirms D1.1 Genotype in dairy cattle in nevada. USDA Animal and Plant Health Inspection Service

[31] CEIRR. 2024. Best practice guidelines for the reporting of milk testing and results, on centers of excellence for influenza research and response. Available from: https://www.ceirr-network.org/news/best-practice-guidelines-for-the-reporting-of-milk-testing-and-results. Retrieved 26 Mar 2025.

[32] Buchholz UJ, Finke S, Conzelmann KK. 1999. Generation of bovine respiratory syncytial virus (BRSV) from cDNA: BRSV NS2 is not essential for virus replication in tissue culture, and the human RSV leader region acts as a functional BRSV genome promoter. J Virol 73:251–259. doi:10.1128/JVI.73.1.251-259.1999 9847328 PMC103829

[33] Schmidt AG, Xu H, Khan AR, O’Donnell T, Khurana S, King LR, Manischewitz J, Golding H, Suphaphiphat P, Carfi A, Settembre EC, Dormitzer PR, Kepler TB, Zhang R, Moody MA, Haynes BF, Liao HX, Shaw DE, Harrison SC. 2013. Preconfiguration of the antigen-binding site during affinity maturation of a broadly neutralizing influenza virus antibody. Proc Natl Acad Sci USA 110:264–269. doi:10.1073/pnas.1218256109 23175789 PMC3538208

[34] McCarthy KR, Lee J, Watanabe A, Kuraoka M, Robinson-McCarthy LR, Georgiou G, Kelsoe G, Harrison SC. 2021. A prevalent focused human antibody response to the influenza virus hemagglutinin head interface. mBio 12:e0114421. doi:10.1128/mBio.01144-21 34060327 PMC8262862

[35] Milder FJ, Jongeneelen M, Ritschel T, Bouchier P, Bisschop IJM, de Man M, Veldman D, Le L, Kaufmann B, Bakkers MJG, Juraszek J, Brandenburg B, Langedijk JPM. 2022. Universal stabilization of the influenza hemagglutinin by structure-based redesign of the pH switch regions. Proc Natl Acad Sci USA 119:e2115379119. doi:10.1073/pnas.2115379119 35131851 PMC8833195

[36] Byrne PO, Fisher BE, Ambrozak DR, Blade EG, Tsybovsky Y, Graham BS, McLellan JS, Loomis RJ. 2023. Structural basis for antibody recognition of vulnerable epitopes on Nipah virus F protein. Nat Commun 14:1494. doi:10.1038/s41467-023-36995-y 36932063 PMC10021056

[37] Simmons HC, Watanabe A, Oguin Iii TH, Van Itallie ES, Wiehe KJ, Sempowski GD, Kuraoka M, Kelsoe G, McCarthy KR. 2023. A new class of antibodies that overcomes a steric barrier to cross-group neutralization of influenza viruses. PLoS Biol 21:e3002415. doi:10.1371/journal.pbio.3002415 38127922 PMC10734940

