# Supplementary material for: Dairy cattle herds mount a characteristic antibody response to highly pathogenic H5N1 avian influenza viruses
Source: J Virol. 2025 Aug 25;99(9):e00621-25. doi: 10.1128/jvi.00621-25 (PMC12455936; doi:10.1128/jvi.00621-25)
Supplement: Supplemental Material — Fig. S1 to S7; Tables S1 and S2. [file jvi.00621-25-s0001.pdf]

Dairy cattle herds mount a characteristic antibody response to highly pathogenic H5N1 avian influenza viruses

Supplementary Material

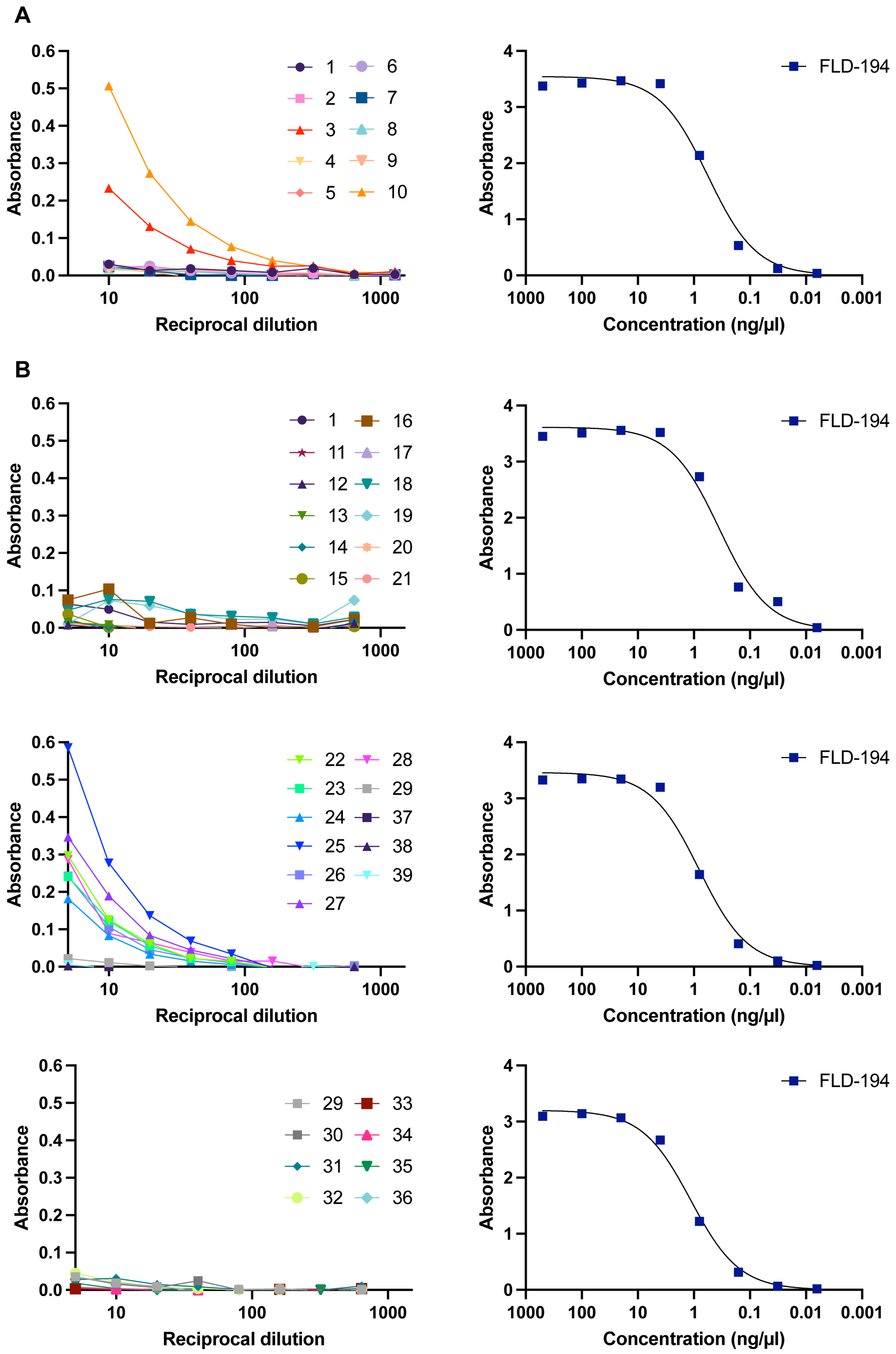

Fig. S1. ELISA screening for H5-reactive antibodies in milk. Milk samples were screened over a range of dilutions for binding to A/dairy cow/Texas/24-008749-001/2024 H5 HA. Background subtracted absorbance values are shown for each plate, alongside standard curves for mAb FLD194 (1) for the same plate. (**A**) Initial California, Colorado, and Pennsylvania samples screened. (**B**) Additional samples from multiple states.

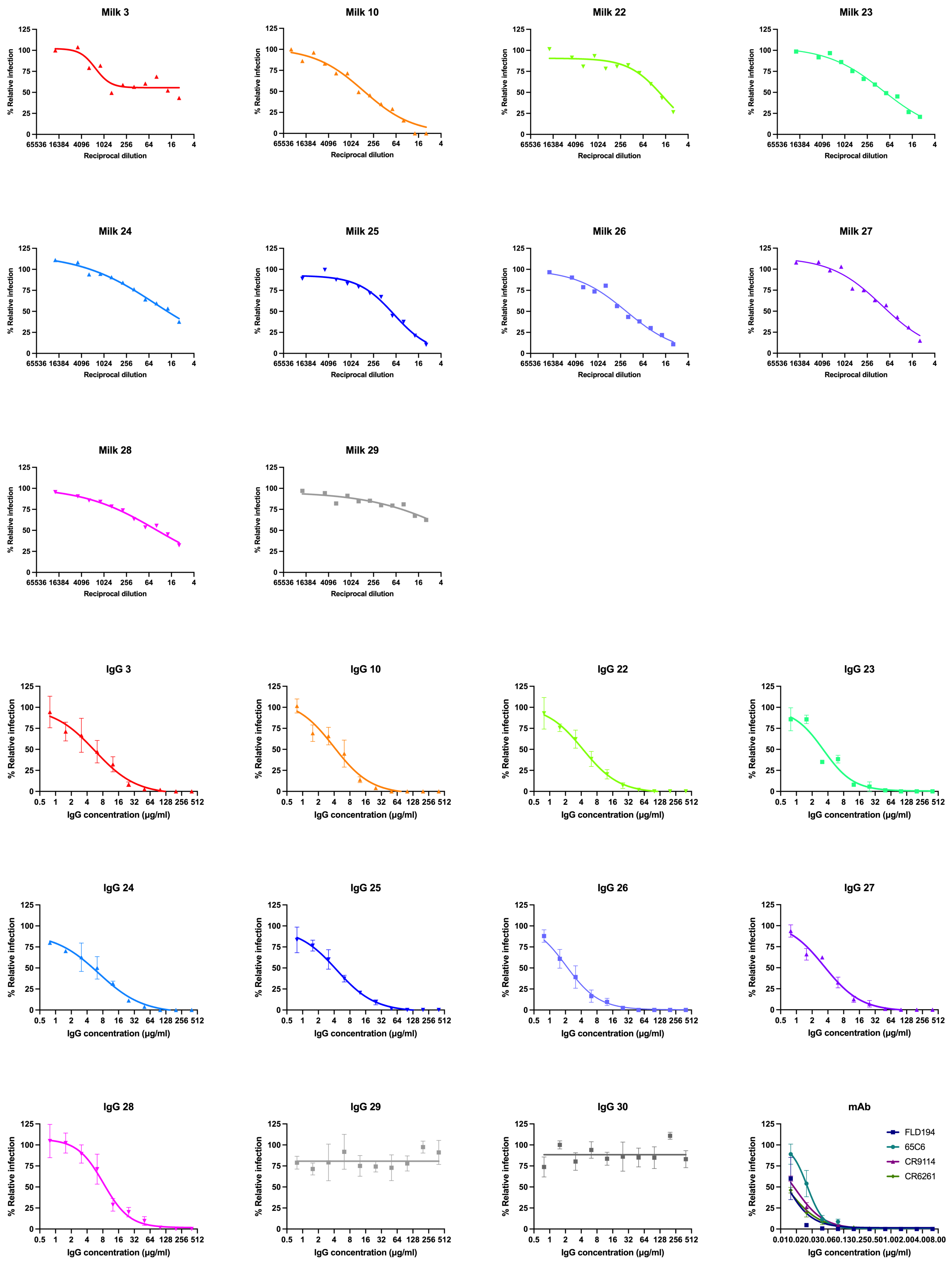

Milk 3
Milk 10
Milk 22
Milk 23
Milk 24
Milk 25
Milk 26
Milk 27
Milk 28
Milk 29
% Relative infection
Reciprocal dilution
65536 16384 4096 1024 256 64 16 4
0 25 50 75 100 125
IgG 3
IgG 10
IgG 22
IgG 23
IgG 24
IgG 25
IgG 26
IgG 27
IgG 28
IgG 29
IgG 30
mAb
IgG concentration (μg/ml)
0.5 1 2 4 8 16 32 64 128 256 512
0.01 0.02 0.03 0.06 0.13 0.25 0.50 1.00 2.00 4.00 8.00
FLD194
65C6
CR9114
CR6261

Fig. S2. Neutralization of rVSV-H5N1dc2024 by milk and purified milk IgG. Fluorescent focus forming neutralization test was performed. Cells infected with rVSV-H5N1dc2024 in the presence of each concentration of milk, purified IgG, or mAbs (1-4) were imaged and fluorescent foci per well were quantified and normalized to wells with no milk/IgG/mAb to determine percent relative infection. Error bars represent standard error of the mean from three independent experiments. Experiments with milk are averages of two independent experiments.

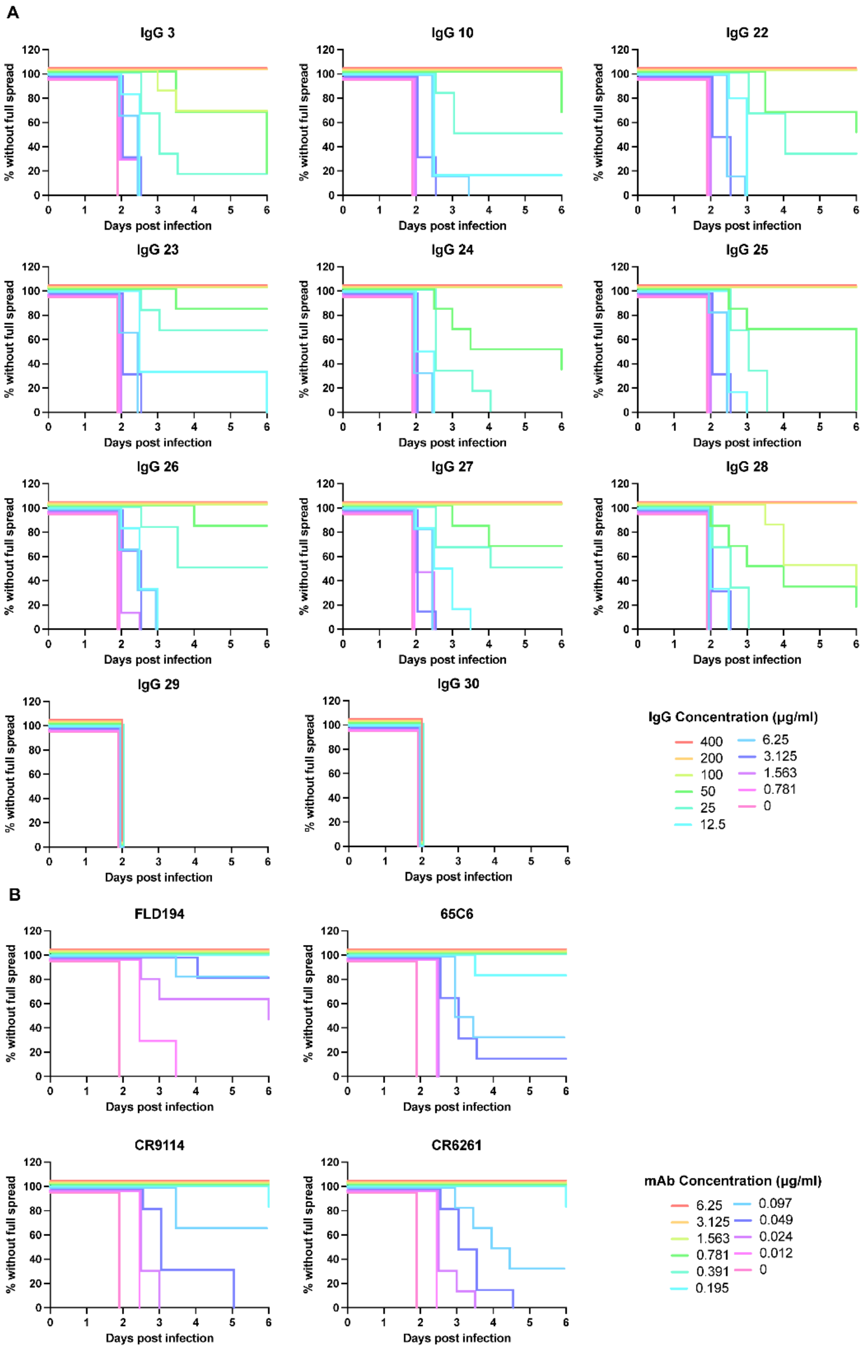

Fig. S3. Purified IgG from milk inhibits viral spread at sub-neutralizing concentrations. (**A**) Spread of rVSV-H5N1dc2024 infection in the presence of purified milk IgG for all samples was assessed twice per day over 5 days for each concentration of IgG. Survival was defined as conditions where infection had not spread throughout the well. Wells which had spreading infection, but which had not reached complete spread by 5 days were annotated as reaching full spread at 6 days. (**B**) The same assay was performed using H5 HA-reactive mAbs FLD194 (1) (HA head binding), 65C6 (2) (HA head binding), CR9114 (3) (HA stem binding), and CR6261 (4) (HA stem binding). Each group represent N=6 total measurements from three independent experiments.

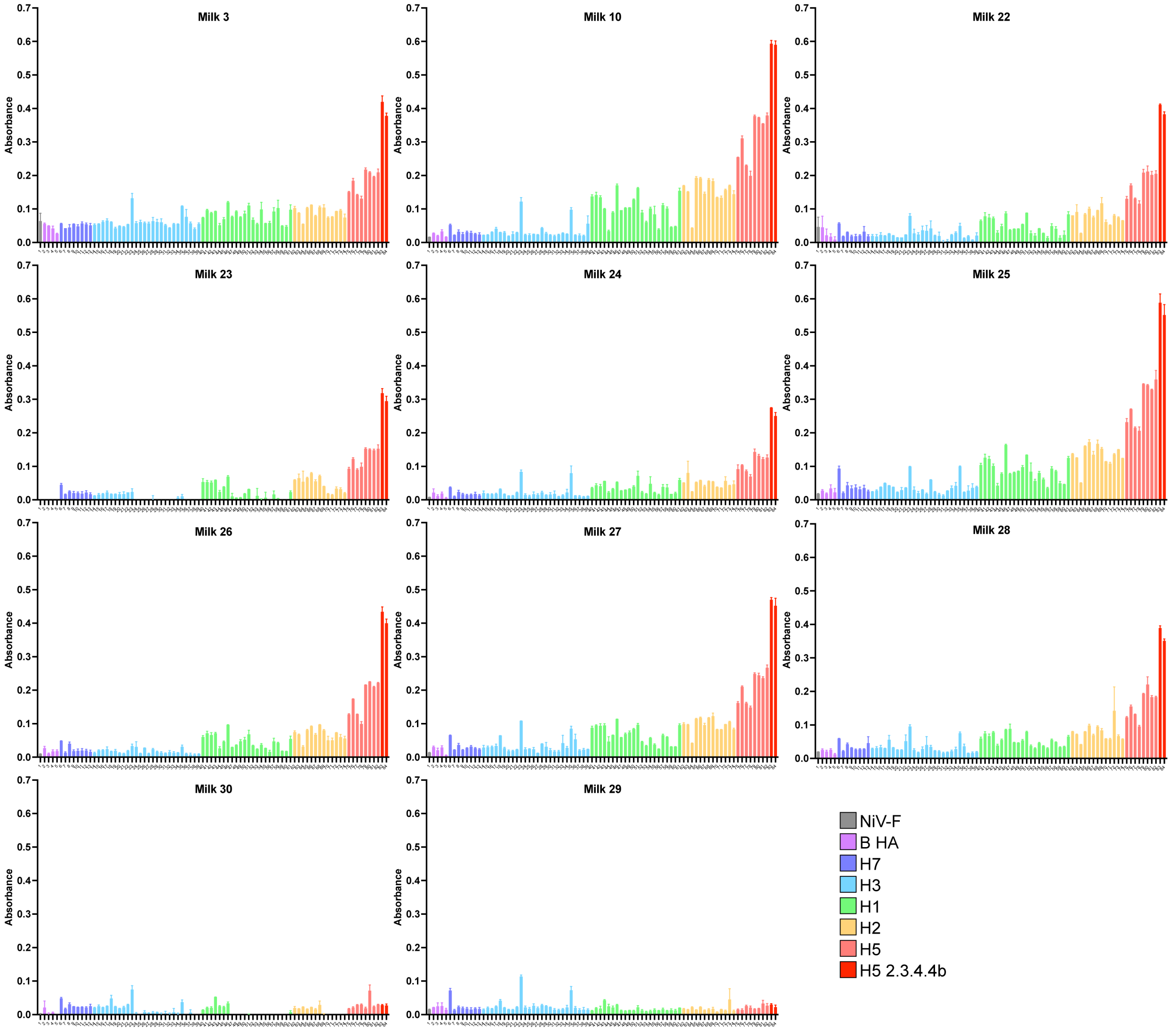

Fig. S4. HA reactivity breadth of milk antibodies. Reactivity of milk antibodies to recombinant full-length secreted ectodomain HA trimers from 83 unique isolates was assessed by ELISA. Nipah virus F protein (NiV-F) was included as a negative control. Bound antibody was detected using HRP-conjugated protein A/G. Bars are colored based on HA subtype, with H5 clade 2.3.4.4.b H5 in darker red for emphasis. The identity of each HA is provided in Table S2.

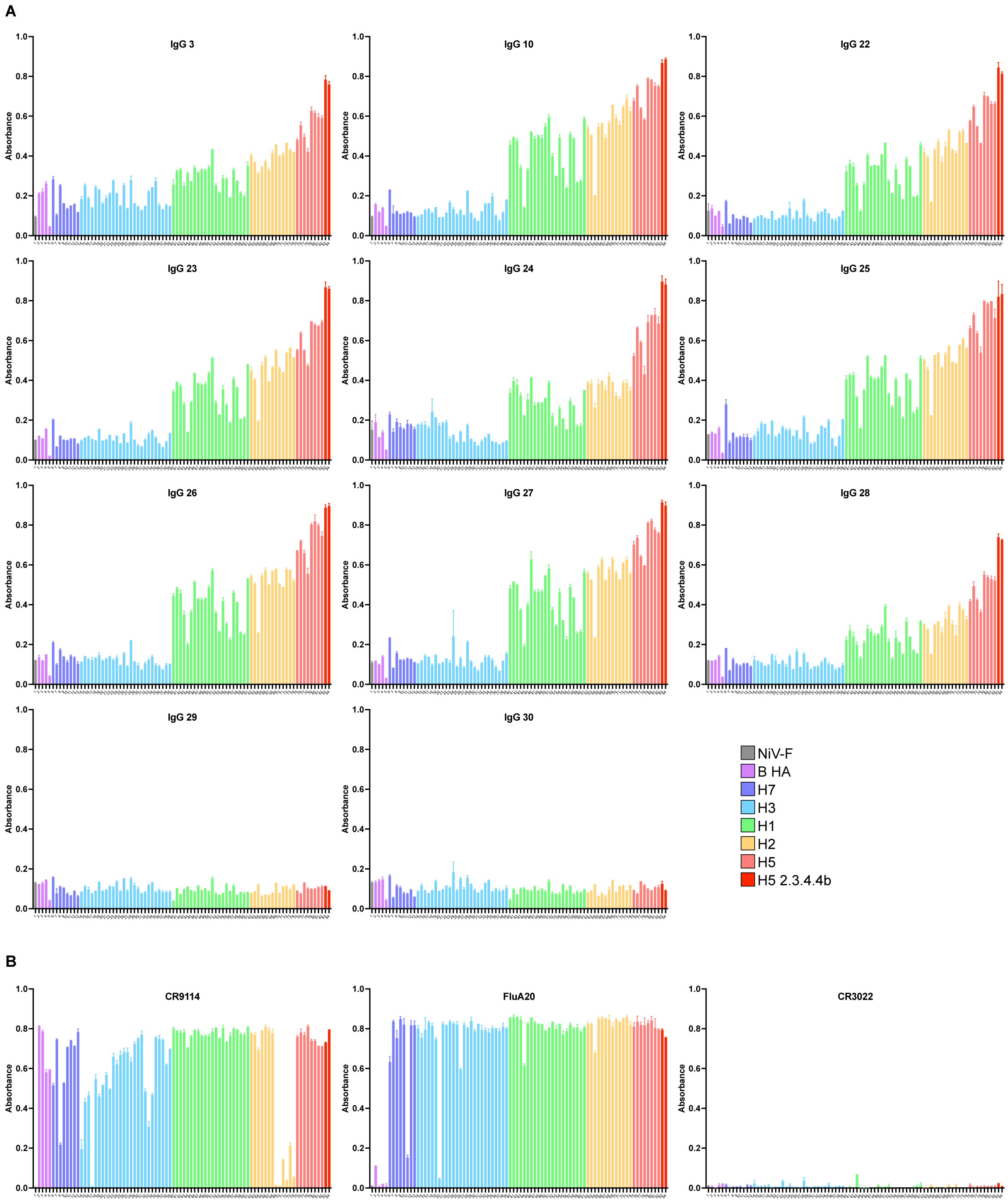

Fig. S5. Breadth of HA reactivity of purified IgG from milk samples. (**A**) Reactivity of purified IgG from milk to recombinant full-length secreted ectodomain HA trimers from 83 unique isolates was assessed by ELISA. Nipah virus F protein (NiV-F) was included as a negative control. Bound antibody was detected using HRP-conjugated protein A/G. Bars are colored based on HA subtype, with H5 clade 2.3.4.4.b H5 in darker red for emphasis. The identity of each HA is provided in Table S2. (**B**) Binding of mAbs CR9114 (3) (broad HA stem binding), FluA-20 (5) (broad HA head binding), and CR3022 (6) (SARS-CoV spike binding) to validate HAs tested using the same ELISA as in (A).

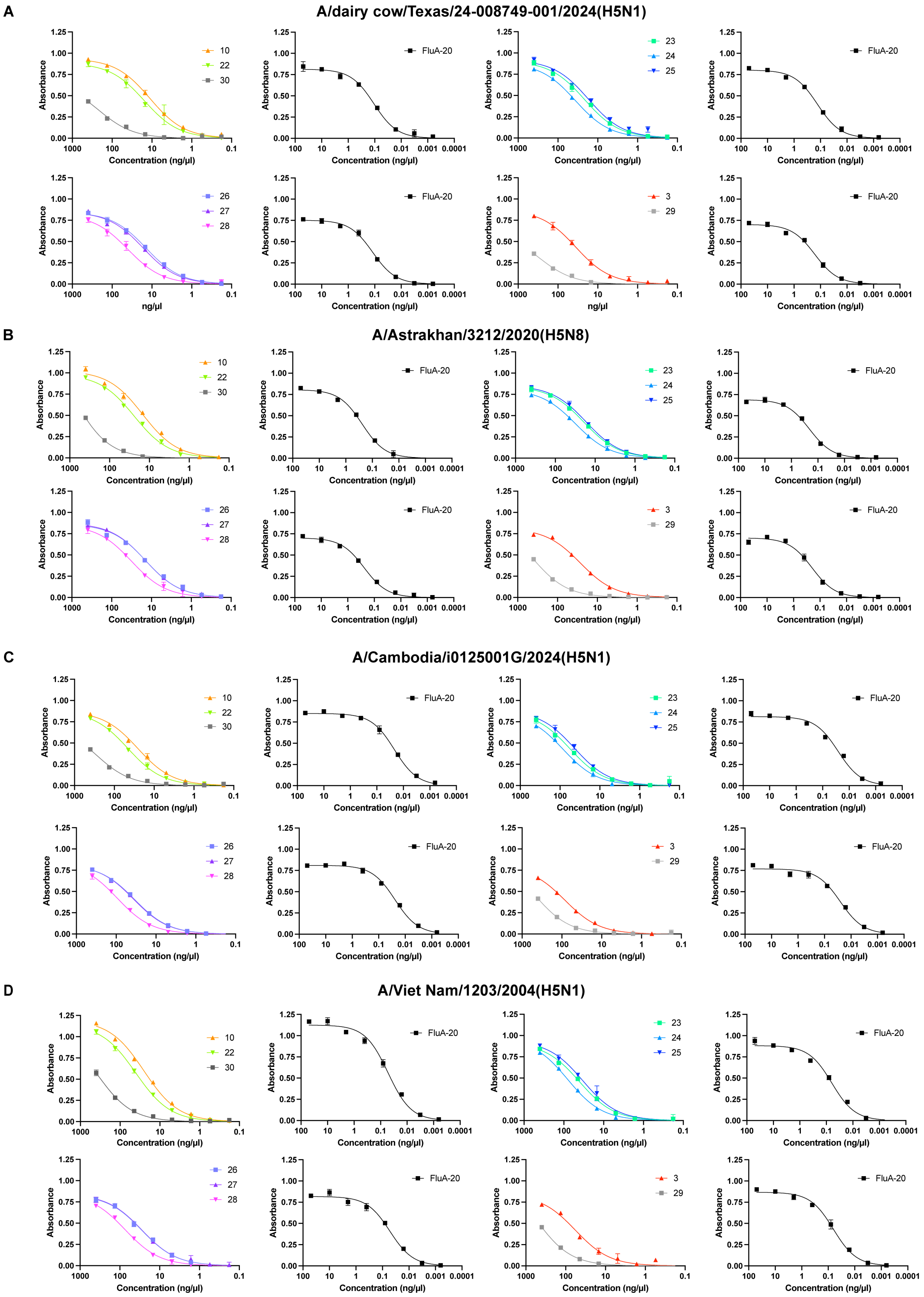
A
A/dairy cow/Texas/24-008749-001/2024(H5N1)
B
A/Astrakhan/3212/2020(H5N8)
C
A/Cambodia/i0125001G/2024(H5N1)
D
A/Viet Nam/1203/2004(H5N1)
Absorbance
Concentration (ng/μl)
ng/μl
10
22
30
23
24
25
26
27
28
3
29
FluA-20

Fig. S6. ELISA titration curves of purified IgG from milk samples to recombinant H5 HAs from the indicated isolates. (**A**) A/dairy cow/Texas/24-008749-001/2024(H5N1), (**B**) A/Astrakhan/3212/2020(H5N8), (**C**) A/Cambodia/i0125001G/2024(H5N1), (**D**) A/Viet Nam/1203/2004(H5N1). Data are background-subtracted absorbance values from three technical replicates. Curves for each ELISA plate are shown alongside standard curves for mAb FluA-20 (5) from the same plate.

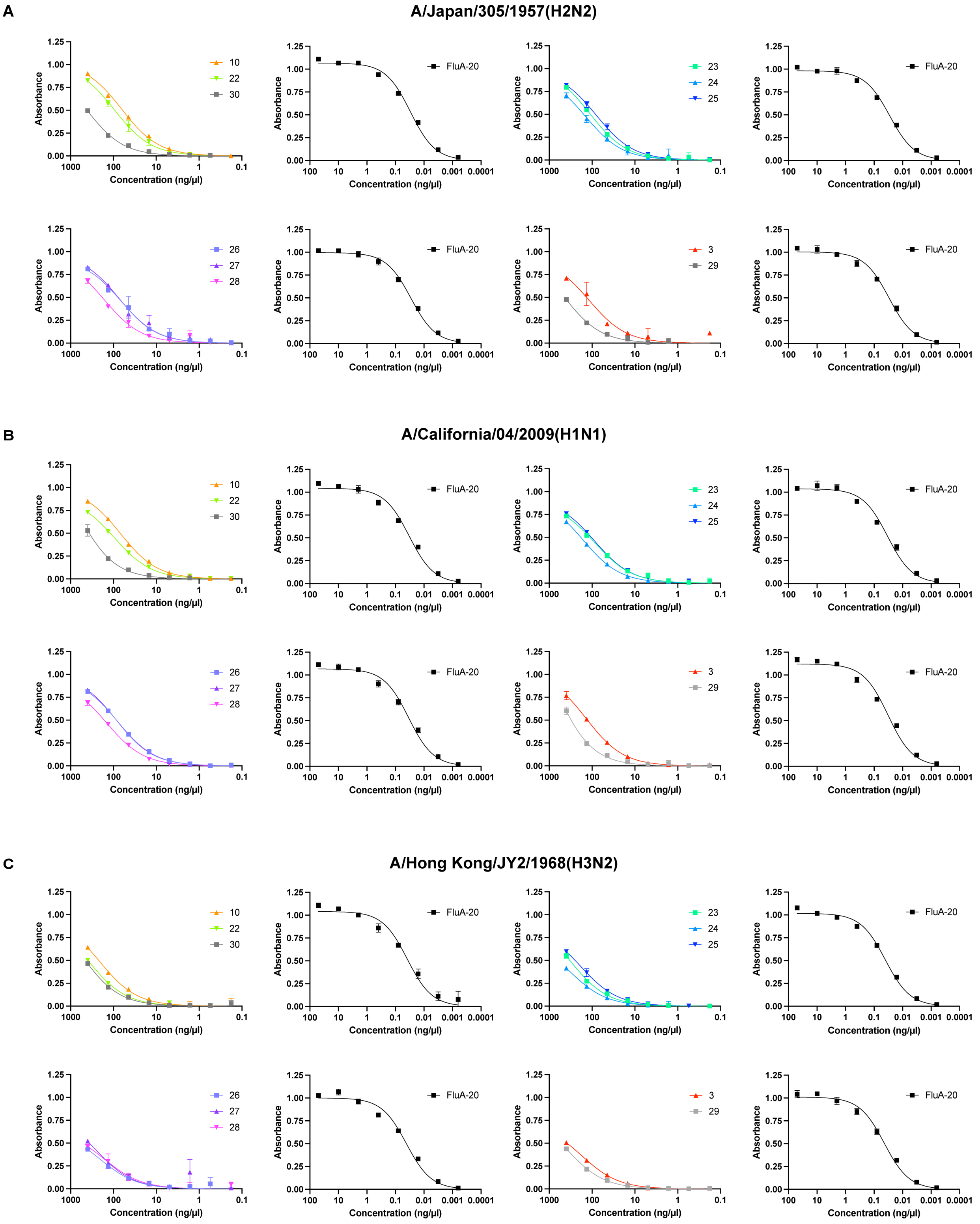

Fig. S7. ELISA titration curves of purified IgG from milk samples to recombinant HAs from the indicated isolates. (**A**) A/Japan/305/1957(H2N2), (**B**) A/California/04/2009(H1N1), (**C**) A/Hong Kong/JY2/1968(H3N2). Data are background subtracted absorbance values from three technical replicates. Curves for each ELISA plate are shown alongside standard curves for mAb FluA-20 (5) from the same plate.

Sample Number	State	Brand alias	Plant alias	Milkfat	Pasteurization	Other processing	Expiration Date	Ab positivity (ELISA)
1	PA	1	1	Whole	Pasteurized		10/22/24	Neg
2	CA	2	2	Skim	Pasteurized		10/22/24	Neg
3	CA	3	2	Skim	Pasteurized		10/15/24	**Pos**
4	CA	4	3	Whole	Pasteurized		10/17/24	Neg
5	CA	5	4	2%	Ultra pasteurized	Lactose Free	11/21/24	Neg
6	CA*	-	-	Cream	-		-	Neg
7	CA*	-	-	Cream	-		-	Neg
8	CA*	-	-	2%	-		-	Neg
9	CO	6	5	2%	Ultra pasteurized		3/7/25	Neg
10	CO	7	6	Whole	Pasteurized		10/21/24	**Pos**
11	CO	8	7	Whole	Ultra pasteurized		12/20/24	Neg
12	CO	9	7	Whole	Ultra pasteurized		12/2/24	Neg
13	CO	9	7	2%	Ultra pasteurized		12/20/24	Neg
14	CA	10	8	2%	Ultra pasteurized		12/10/24	Neg
15	CA	10	9	Whole	Vat pasteurized		-	Neg
16	CA	8	10	-	-		-	Neg
17	CA	11	11	Whole	Pasteurized		10/23/24	Neg
18	CA	8	3	Whole	Pasteurized		10/31/24	Neg
19	MN	8	12	Whole	Pasteurized		11/3/24	Neg
20	MI	12	13	Whole	Ultra pasteurized	Lactose free, ultra filtered	12/18/24	Neg
21	OH	13	14	2%	Ultra pasteurized		10/29/24	Neg
22	CO	14	5	Skim	Pasteurized		11/23/24	**Pos**
23	CO	14	5	Whole	Pasteurized		11/28/24	**Pos**
24	CO	15	15	2%	Pasteurized		11/20/24	**Pos**
25	CO	16	6	2%	Pasteurized		11/18/24	**Pos**
26	CO	17	16	Whole	Pasteurized		11/22/24	**Pos**
27	CO	18	6	2%	-	Lactose Free	11/10/24	**Pos**
28	CO	7	6	Whole	Pasteurized		11/20/24	**Pos**
29	PA	19	17	Whole	Pasteurized		11/27/24	Neg
30	PA	1	1	Whole	Pasteurized		11/29/24	Neg
31	MI	6	18	Whole	Pasteurized		12/5/24	Neg
32	MI	6	18	1%	Pasteurized		12/4/24	Neg
33	UT	20	19	Whole	Ultra pasteurized		12/2/24	Neg
34	MI	12	18	Whole	Ultra pasteurized	Lactose free, ultra filtered	1/14/25	Neg
35	KS	3	20	Whole	Ultra pasteurized		1/17/25	Neg
36	CO	6	5	Half & Half	Ultra pasteurized		3/9/25	Neg

Table S1. Complete information for milk samples. "-" information was unavailable. "*" Samples 6-8 are presumed to be from California, but brand and processing plant information were unavailable. Antibody positivity was determined by ELISA with A/dairy cow/Texas/24-008749-001/2024 H5 HA (See Fig. S1). Brand and processing plants were deidentified and provided with numerical aliases.

Number	Subtype	Isolate	Accession	Stabilized
1		Nipah F		+
2	B	B/Phuket/3073/2013	EPI540671	
3	B	B/Brisbane/60/2008	CY115151	
4	B	B/Florida/04/2006	EU515992	
5	B	B/Lee/1940	DQ792897	
6	H7	A/chicken/Durango/CPA-03739-22/2022(H7N3)	EPI_ISL_16209257	
7	H7	A/ruddy turnstone/Delaware/199/2020(H7N3)	EPI_ISL_4061233	
8	H7	A/Gansu/23277/2019(H7N9)	EPI_ISL_353997	
9	H7	A/Taiwan/01/2017(H7N9)	EPI917065	
10	H7	A/goose/Jiangsu/1027/2013(H7N9)	EPI_ISL_173693	
11	H7	A/New York/107/2003(H7N2)	EU587368	
12	H7	A/Netherlands/33/2003(H7N7)	EPI_ISL_3545	
13	H7	A/England/268/1996(H7N7)	EPI_ISL_952	
14	H3	A/China/ZMD-22-2/2022(H3N8)	EPI_ISL_15613648	
15	H3	A/equine/Kentucky/03/2021(H3N8)	EPI_ISL_15604680	
16	H3	A/Hawaii/28/2020(H3N2v)	MW024914	
17	H3	A/swine/Italy/102079/2018(H3N2)	EPI_ISL_14751284	
18	H3	A/Iowa/38/2017(H3N2v)	MK239073	
19	H3	A/feline/Korea/FY057/2014(H3N2)	EPI_ISL_279527	+
20	H3	A/swine/Ohio/12TOSU293/2012(H3N2)	JX534969	
21	H3	A/duck/Chiba/24/2006(H3N8)	EPI_ISL_291120	
22	H3	A/seal/Massachusetts/3911/1992(H3N3)	EPI_ISL_68832	
23	H3	A/Darwin/6/2021(H3N2)	EPI_ISL_1563628	
24	H3	A/Tasmania/503/2020(H3N2)	EPI_ISL_483574	
25	H3	A/Kansas/14/2017(H3N2)	EPI_ISL_292575	
26	H3	A/Hong Kong/4801/2014(H3N2)(X-263B)	EPI765207	+
27	H3	A/Hong Kong/4801/2014(H3N2)	EPI675798	
28	H3	A/Texas/50/2012(H3N2)	KJ942616	
29	H3	A/Perth/16/2009(H3N2)	EPI_ISL_31055	
30	H3	A/Wisconsin/67/2005(H3N2)	EU103823	+
31	H3	A/Fujian/411/2002(H3N2)	EPI_ISL_167284	+
32	H3	A/Moscow/10/1999(H3N2)	DQ487341	
33	H3	A/Sydney/05/1997(H3N2)	EF566075	
34	H3	A/Johannesburg/33/1994(H3N2)	CY121341	
35	H3	A/Beijing/353/1986(H3N2)	EPI_ISL_123212	+
36	H3	A/Leningrad/360/1986(H3N2)	EPI_ISL_937	+
37	H3	A/Philippines/02/1983(H3N2)	CY113301	+
38	H3	A/Victoria/03/1975(H3N2)	V01098	
39	H3	A/Hong Kong/JY2/1968(H3N2)	CY147438	
40	H1	A/England/234600203/2023(H1N2v)	EPI_ISL_18548251	
41	H1	A/swine/Texas/A02245420/2020(H1N2)	MT232791	
42	H1	A/swine/Minnesota/A02245409/2020(H1N1)	MT154189	
43	H1	A/Michigan/383/2018(H1N2v)	MK239081	
44	H1	A/Ontario/N163578/2012(H1N1v)	JX875001	+
45	H1	A/Delaware/300/2009(H1N1)	KF424130	
46	H1	A/New Jersey/08/1976(H1N1v)	CY039991	
47	H1	A/West Virginia/30/2022(H1N1)	EPI2756244	
48	H1	A/Brisbane/02/2018(H1N1)	EPI_ISL_306350	
49	H1	A/Michigan/45/2015(H1N1)	EPI_ISL_199532	
50	H1	A/California/7/2009(H1N1)(X181)	GQ906801	
51	H1	A/California/04/2009(H1N1)	FJ966082	
52	H1	A/Solomon Islands/03/2006(H1N1)	EU100724	
53	H1	A/New Caledonia/20/1999(H1N1)	CY033622	
54	H1	A/Texas/36/1991(H1N1)	CY033655	
55	H1	A/Singapore/6/1986(H1N1)	CY020477	
56	H1	A/Chile/01/1983(H1N1)	EPI_ISL_125876	+
57	H1	A/USSR/90/1977(H1N1)	EPI_ISL_66104	
58	H1	A/Denver/1/1957(H1N1)	CY008988	
59	H1	A/Fort Monmouth/01/1947(H1N1)	CY045780	+
60	H1	A/Puerto Rico/08/1934(H1N1)	NC_002017	+
61	H1	A/South Carolina/1/1918(H1N1)	AF117241	
62	H2	A/swan/Poland/MB152/2022(H2N3)	EPI_ISL_18245819	
63	H2	A/snow goose/Delaware/12OS3778/201(H2N3)	EPI_ISL_216102	
64	H2	A/mallard/Minnesota/AI08-3437/2008(H2N3)	CY141160	+
65	H2	A/swine/Missouri/2124514/2006(H2N3)	EPI_ISL_13851	
66	H2	A/duck/Hokkaido/W259/2005(H2N5)	EPI_ISL_293517	
67	H2	A/ruddy turnstone/Delaware/142/1998(H2N8)	EPI_ISL_99393	
68	H2	A/mallard/Potsdam/179/1983(H2N2)	EPI_ISL_118683	
69	H2	A/North Carolina/01/1968(H2N2)	EPI_ISL_19109	
70	H2	A/Berkeley/01/1968(H2N2)	EPI_ISL_235	
71	H2	A/Johannesburg/617/1967(H2N2)	EPI_ISL_19669	
72	H2	A/Cornell/1001/1967(H2N2)	EPI_ISL_130363	
73	H2	A/Netherlands/056H1/1960(H2N2)	EPI_ISL_84895	
74	H2	A/Japan/305/1957(H2N2)	CY014976	
75	H5	A/turkey/Ontario/7732/1966(H5N9)	CY107859	
76	H5	A/turkey/England/N28/1973(H5N2)	GU052548	
77	H5	A/ostrich/Denmark/96-72420/1996(H5N2)	EPI_ISL_29260	
78	H5	A/State of Mexico/INER-INF645/2024(H5N2)	EPI_ISL_19186450	
79	H5	A/Hong Kong/156/1997(H5N1)	AF046088	
80	H5	A/Viet Nam/1203/2004(H5N1)	AY818135	
81	H5	A/Egypt/N03072/2010(H5N1)	CY062484	
82	H5	A/Cambodia/i0125001G/2024(H5N1)	EPI_ISL_18823967	
83	H5	A/Astrakhan/3212/2020(H5N8)	EPI_ISL_1038924	
84	H5	A/dairy cow/Texas/24-008749-001/2024(H5N1)	EPI_ISL_19014384	+

Table S2. Information for recombinant HAs used for ELISAs. Nipah F was used a negative control. HAs indicated with a "+" contain stabilizing mutations (7, 8).

REFERENCES

1. Xiong X, Corti D, Liu J, Pinna D, Foglierini M, Calder LJ, Martin SR, Lin YP, Walker PA, Collins PJ, Monne I, Suguitan AL, Jr., Santos C, Temperton NJ, Subbarao K, Lanzavecchia A, Gamblin SJ, Skehel JJ. 2015. Structures of complexes formed by H5 influenza hemagglutinin with a potent broadly neutralizing human monoclonal antibody. Proc Natl Acad Sci U S A 112:9430-5.
2. Hu H, Voss J, Zhang G, Buchy P, Zuo T, Wang L, Wang F, Zhou F, Wang G, Tsai C, Calder L, Gamblin SJ, Zhang L, Deubel V, Zhou B, Skehel JJ, Zhou P. 2012. A human antibody recognizing a conserved epitope of H5 hemagglutinin broadly neutralizes highly pathogenic avian influenza H5N1 viruses. J Virol 86:2978-89.
3. Dreyfus C, Laursen NS, Kwaks T, Zuijdgeest D, Khayat R, Ekiert DC, Lee JH, Metlagel Z, Bujny MV, Jongeneelen M, van der Vlugt R, Lamrani M, Korse HJ, Geelen E, Sahin O, Sieuwerts M, Brakenhoff JP, Vogels R, Li OT, Poon LL, Peiris M, Koudstaal W, Ward AB, Wilson IA, Goudsmit J, Friesen RH. 2012. Highly conserved protective epitopes on influenza B viruses. Science 337:1343-8.
4. Ekiert DC, Bhabha G, Elsliger MA, Friesen RH, Jongeneelen M, Throsby M, Goudsmit J, Wilson IA. 2009. Antibody recognition of a highly conserved influenza virus epitope. Science 324:246-51.
5. Bangaru S, Lang S, Schotsaert M, Vanderven HA, Zhu X, Kose N, Bombardi R, Finn JA, Kent SJ, Gilchuk P, Gilchuk I, Turner HL, Garcia-Sastre A, Li S, Ward AB, Wilson IA, Crowe JE, Jr. 2019. A Site of Vulnerability on the Influenza Virus Hemagglutinin Head Domain Trimer Interface. Cell 177:1136-1152 e18.
6. ter Meulen J, van den Brink EN, Poon LL, Marissen WE, Leung CS, Cox F, Cheung CY, Bakker AQ, Bogaards JA, van Deventer E, Preiser W, Doerr HW, Chow VT, de Kruif J, Peiris JS, Goudsmit J. 2006. Human monoclonal antibody combination against SARS coronavirus: synergy and coverage of escape mutants. PLoS Med 3:e237.
7. Milder FJ, Jongeneelen M, Ritschel T, Bouchier P, Bisschop IJM, de Man M, Veldman D, Le L, Kaufmann B, Bakkers MJG, Juraszek J, Brandenburg B, Langedijk JPM. 2022. Universal stabilization of the influenza hemagglutinin by structure-based redesign of the pH switch regions. Proc Natl Acad Sci U S A 119.
8. Byrne PO, Fisher BE, Ambrozak DR, Blade EG, Tsybovsky Y, Graham BS, McLellan JS, Loomis RJ. 2023. Structural basis for antibody recognition of vulnerable epitopes on Nipah virus F protein. Nat Commun 14:1494.
